# Human Mind Control of Rat Cyborg’s Continuous Locomotion with Wireless Brain-to-Brain Interface

**DOI:** 10.1038/s41598-018-36885-0

**Published:** 2019-02-04

**Authors:** Shaomin Zhang, Sheng Yuan, Lipeng Huang, Xiaoxiang Zheng, Zhaohui Wu, Kedi Xu, Gang Pan

**Affiliations:** 1 Qiushi Academy for Advanced Studies (QAAS), Zhejiang University, Hangzhou, China; 2 Department of Computer Science, Zhejiang University, Hangzhou, China; 3 Department of Biomedical Engineering, Key Laboratory of Biomedical Engineering of Education Ministry, Zhejiang University, Hangzhou, China; 4 Zhejiang Provincial Key Laboratory of Cardio-Cerebral Vascular Detection Technology and Medicinal Effectiveness Appraisal, Zhejiang University, Hangzhou, China

## Abstract

Brain-machine interfaces (BMIs) provide a promising information channel between the biological brain and external devices and are applied in building brain-to-device control. Prior studies have explored the feasibility of establishing a brain-brain interface (BBI) across various brains via the combination of BMIs. However, using BBI to realize the efficient multidegree control of a living creature, such as a rat, to complete a navigation task in a complex environment has yet to be shown. In this study, we developed a BBI from the human brain to a rat implanted with microelectrodes (i.e., rat cyborg), which integrated electroencephalogram-based motor imagery and brain stimulation to realize human mind control of the rat’s continuous locomotion. Control instructions were transferred from continuous motor imagery decoding results with the proposed control models and were wirelessly sent to the rat cyborg through brain micro-electrical stimulation. The results showed that rat cyborgs could be smoothly and successfully navigated by the human mind to complete a navigation task in a complex maze. Our experiments indicated that the cooperation through transmitting multidimensional information between two brains by computer-assisted BBI is promising.

## Introduction

Direct communication between brains has long been a dream for people, especially for those with difficulty in verbal or physical language. Brain-machine interfaces (BMIs) provide a promising information channel between the brain and external devices. As a potential human mind reading technology, many previous BMI studies have successfully decoded brain activity to control either virtual objects^1–3^ or real devices^4,5^. On the other hand, BMIs can also be established in an inverse direction of information flow, where computer-generated information can be used to modulate the function of a specific brain region^6–8^ or import tactile information back to the brain^9–11^. The combination of different types of BMI systems can thus help to realize direct information exchange between two brains to form a new brain-brain interface (BBI). However, very few previous studies have explored BBIs across different brains^12^. Miguel Pais-Vieira *et al*. established a BBI to realize the real-time transfer of behaviorally meaningful sensorimotor information between the brains of two rats^13^. While an encoder rat performed a sensorimotor task, samples of its cortical activity were transmitted to matching cortical areas of a “decoder” rat using intracortical micro-electrical stimulation (ICMS) on its somatosensory cortex. Guided solely by the information provided by the encoder rat’s brain, the decoder rat learned to make similar behavioral selections. BBIs between humans have also been preliminary explored. One example of a BBI between humans detected motor intention with EEG signals recorded from one volunteer and transmitted this information over the internet to the motor cortex region of another volunteer by transcranial magnetic stimulation, which resulted in the direct information transmission from one human brain to another using noninvasive means^14^. In addition to information transfer between two brains of the same type of organism, the BBI enables information to be transferred from a human brain to another organism’s brain. Yoo *et al*. used steady-state visual evoked potential (SSVEP)-based BMI to extract human intention and sent it to an anesthetized rat using transcranial focused ultrasound stimulation on its brain, thereby controlling the tail movement of the anesthetized rat by the human brain^15^. In a very recent work, a BBI was developed to implement motion control of a cyborg cockroach by combining a human’s SSVEP BMI and electrical nerve stimulation on the cockroach’s antennas^16^. The cyborg cockroach could then be navigated by the human brain to complete walking along an S-shaped track.

Although the feasibility of BBIs has been preliminarily proven, it is still a big challenge to build an efficient BBI for the multidegree control for the continuous locomotion of a mammal in a complex environment. In the current study, we present a wireless brain-to-brain interface, through which a human can mind control a live rat’s continuous locomotion. Different from the control of lifeless devices, it is critical to have highly demanding instantaneity in the control of a living creature in real time due to its agility and self-consciousness. For this purpose, the BBI system requires timely reactions and a high level of accuracy in terms of information decoding and importing, as well as real-time visual feedback of the rat’s movement. The SSVEP-based BMI, as used for brain intention decoding in previous BBI works that have depended on visual stimulation, may distract the manipulator from reacting promptly to real-time visual feedback. As an alternative solution, motor imagery-based BMI has the advantages of rapid response and a low level of distraction from the visual feedback. Therefore, the BBI system established in the current study integrates control instructions decoded by noninvasive motor imagery with neural feedback, and the instructions are sent back to the rat’s brain by ICMS in real time. We also proposed and compared two different control models for our BBI system, the thresholding model (TREM) and the gradient model (GRAM), to provide a more natural and easier process for the manipulator during steering control. With this interface, our manipulators were able to mind control a rat cyborg to smoothly complete maze navigation tasks.

## Results

### Set up of BBI system and task design

**Figure 1:**
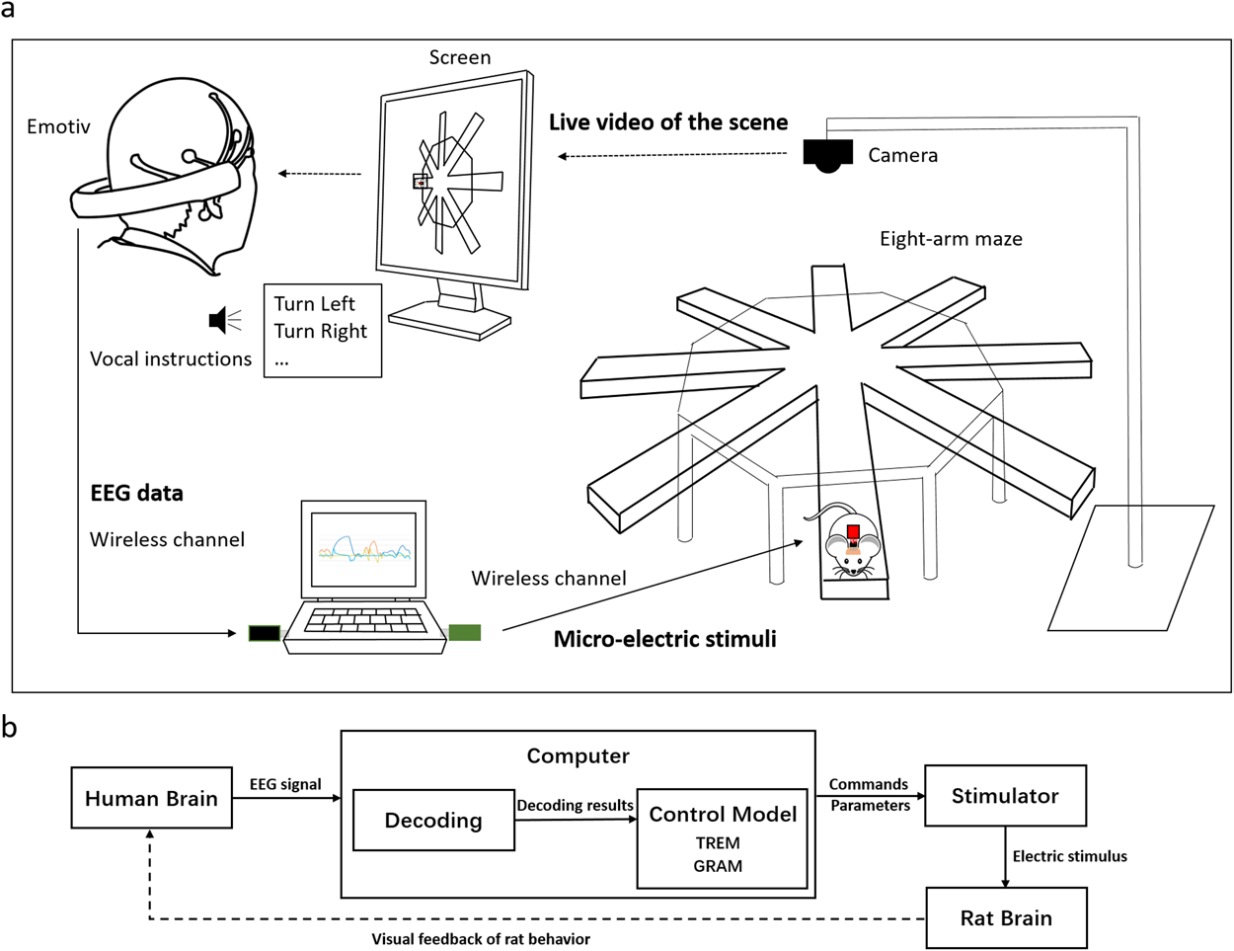
Experiment setup. (**a**) Overview of the BBI system. In the brain control sessions, EEG signal was acquired and sent to the host computer where the motor intent was decoded. The decoding results were then transferred into control instructions and sent to the stimulator on the back of the rat cyborg with preset parameters. The rat cyborg would then respond to the instructions and finish the task. For the eight-arm maze, the width of each arm was 12 cm and the height of the edge was 5 cm. The rat cyborg was located in the end of either arm at the beginning of each run. And preset turning directions were informed vocally by another participant when a new trial started. (**b**) Flowchart of the proposed brain-to-brain interface.

The BBI system in the current study consisted of two parts: a noninvasive EEG-based BMI and a rat cyborg system^17^ (Fig. 1(a)). The EEG-based BMI decoded the motor intent of left and right arm movement, which corresponded to the generation of instruction *Left* and *Right* turning, respectively. In the current study, the average EEG signal control accuracy of all 6 manipulators was 77.86 ± 12.4% over all the experiments conducted. The eye blink signals in the EEG were used to elicit the instruction *Forward/Reward*, which was detected by the amplitude of EEG signal in the frontopolar channel. The rat cyborgs were prepared based on previous works^17–20^ and were well-trained before experiments were conducted in this study (see Methods for more details). Two parts of the system were connected through an integration platform, sending decoded instructions from motor intent to the rat cyborgs, and providing visual information feedback in real time. An overview of the BBI system is presented in Fig. 1.

The control effect of the rat cyborgs was evaluated by a turning task on an eight-arm maze. A complete run of the turning task contained a total of 16 turning trials, with eight left turnings and eight right turnings. To avoid the influence of the memory and training experience of the rats, the turning direction sequence was randomly generated by computer before each task run. The targeted turning direction of each trial was informed vocally by other experimenters at the start of each trial during the turning control experiments. For each run, the rats were placed at the end of one of the eight arms as a starting point. The rat was then driven towards the center of the maze and guided to turn into one of the adjacent arms. A trial was regarded as successful when the rat performed a correct turning and reached the end of the target arm. A new trial would then start when the rat reached the end of one arm and turned its head back towards the center of the maze. If the rat failed to complete one turning trial, the same turning direction trail was repeated until the rat succeeded. The total time from the start to the end of completing 16 correct trials was recorded as the completion time (CPT) of each run. The turning accuracy (TA) was then calculated as the ratio of the number of correct turns to the total number of turns performed.

The entire experiment contained three stages, one manual control stage and two brain control stages, with each stage containing 5 sessions and being performed on five consecutive days. Each session consisted of 3 independent runs, with an interval break time between each run of at least eight minutes. The entire procedure was video recorded, and the mouse clicking sequences during manual control stage were recorded for further analysis. In the second and third stages, two different control models (GRAM and TREM, see details in the Methods) were applied. To further test the applicability of brain control, the rat cyborgs were controlled to complete a navigation task in a more complicated maze.

### Manual control of rat cyborg

**Figure 2:**
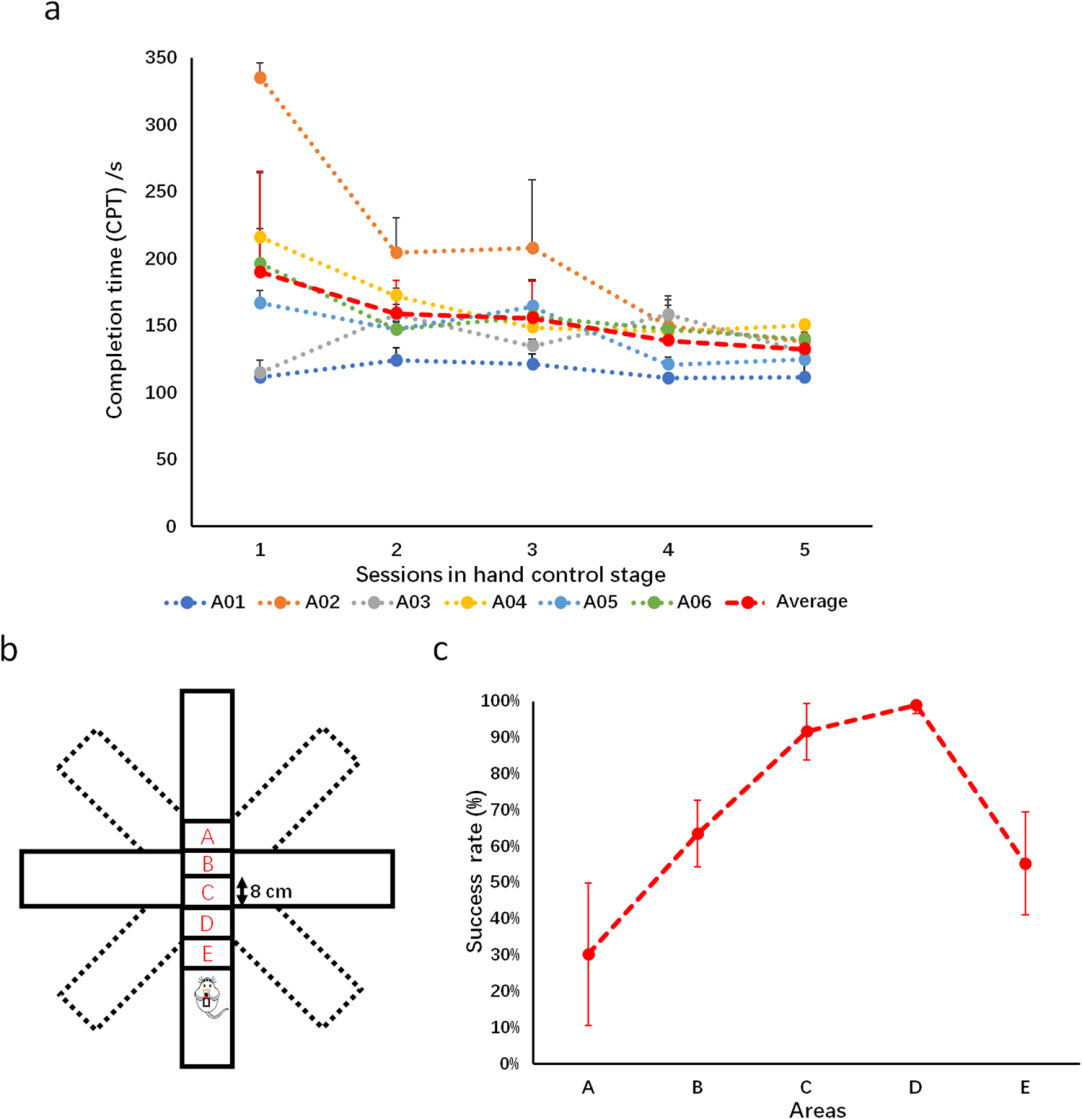
(**a**) Performance of manual control stage. The mean CPT of each rat cyborg for manual control across all sessions. (Note: For display, only positive standard deviations are presented as error bars). (**b**) Different areas assigned in the investigation for the optimal area. The simplified plus-maze was modified from the original eight-arm maze by blocking four crossing arms. (**c**) The averaged success rate (mean ± SD) of each area for the rat cyborgs to receive instructions with manual control.

During the manual control stage, the rat cyborgs were controlled by experienced operators. We found that the turning accuracy of a well-trained rat cyborg could achieve an exceptionally high rate of nearly 100%. As displayed in Fig. 2(a), the average CPT of all rat cyborgs at the first session of manual control was 190.03 ± 75.41 s and decreased to 132.56 ± 12.39 s at the fifth session. Most of the rats showed an obvious learning curve through the manual control stage. The CPT of each rat cyborg became very close at the end of the manual control stage, indicating that they were becoming familiar with the task environment and the control instructions delivered into their brains. There was no significant difference (paired T-test, p > 0.05) between the average CPT of the last two sessions of the manual control stage for each rat cyborg, which indicated that the rat cyborgs were in a steady state.

During the manual control sessions, we noticed that the successful turning behavior of a rat cyborg was highly dependent on the timing of the turning instructions (Fig. 2(b)). To optimize the instruction timing, an additional experiment was conducted. In this experiment, the rats were placed at the end of the plus-maze, which was modified from the original eight-arm maze, to wait for instructions to turn left or right. By delivering turning instructions while the rats’ bodies were located in different sections along the straight arm, the instruction timing could be evaluated by the turning success of the rats. Figure 2(c) shows the overall performance of the turning success rate at five equally divided sections of the maze. According to the success rate of this plus-maze test, the best location for the rat cyborg to receive turning instructions was the area near the intersection (areas C and D in Fig. 2(b)). When considering brain control conditions, motor imagery should be initiated slightly before the optimal point for manual control because the decoding process and instruction generation take a short period of time. Thus, in our study, the manipulators were asked to start motor imagery when the rats arrived at areas D and E.

### BBI evaluation

**Figure 3:**
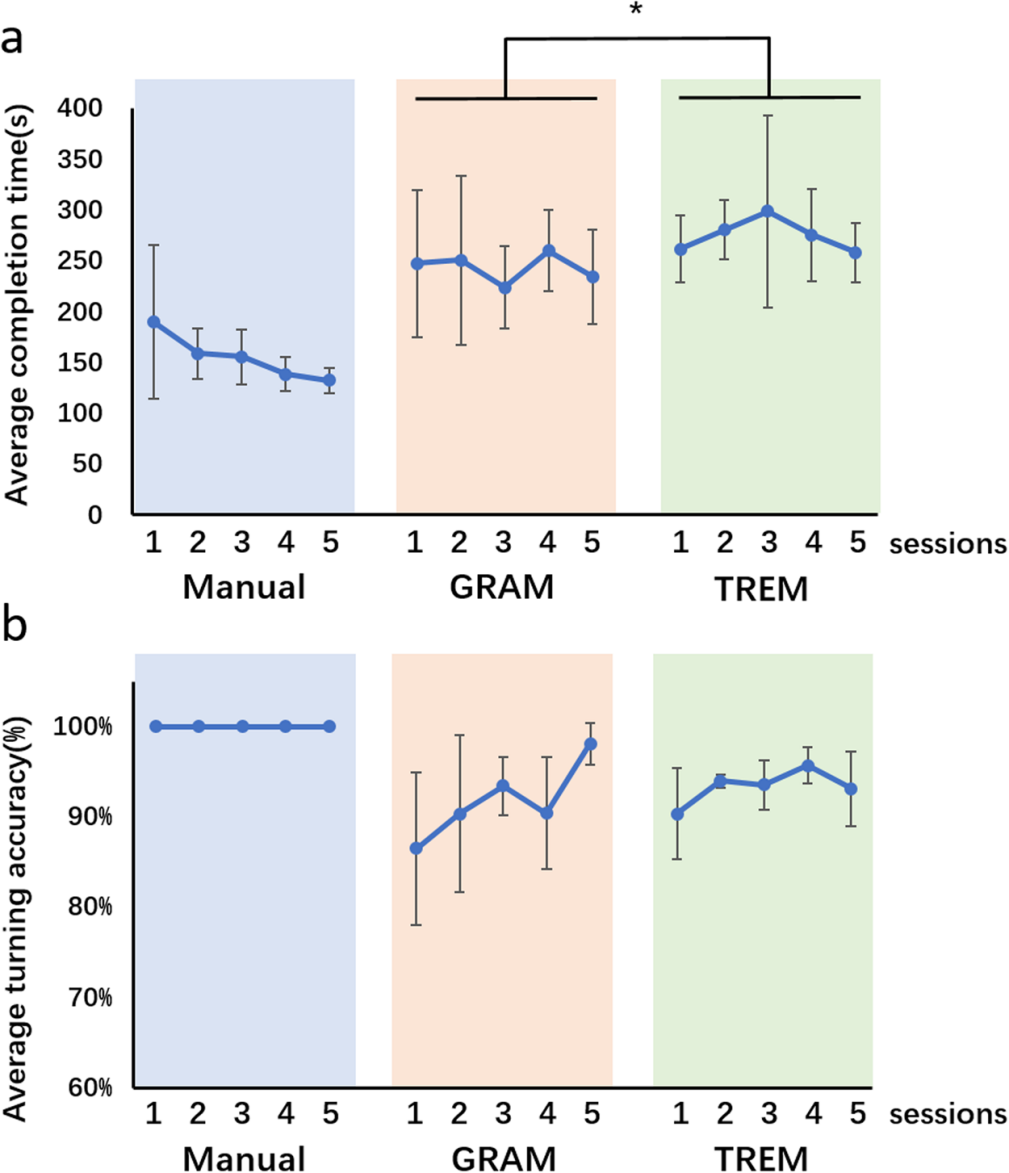
(**a**) Average CPT across all rat cyborgs for the three consecutive stages. (**b**) Average turning accuracy across all rat cyborgs for the three consecutive stages. Error bars indicate the standard deviation. *Indicates p < 0.05.

After stage 1 of manual control, two further brain control stages were performed by several brain control manipulators. In the two brain control stages, the manipulators controlled the rat cyborgs with a BBI (Fig. 1(a)) based on one of the two proposed control models. During the first brain control stage (stage 2), the gradient model (GRAM) was applied, and in the second brain control stage, the thresholding model (TREM) was applied. The two control models were based on different threshold calculating strategies. The thresholds were used to differentiate the decoding results attributed to real intention or noise (see Methods for a detailed explanation of thresholds). The results of the two control models are shown in Fig. 3. The overall CPT value remained stable in both brain control stages, with no significant difference between the two sessions inside each stage (Fig. 3(a), paired T-test for the average CPT, p > 0.05). However, a comparison between the two brain control stages showed that a longer time was taken to complete the same maze tasks with the TREM-based BBI system. The average CPT of all rat cyborgs across the GRAM-based stage 2 was shorter than the TREM-based stage 3 (243.41 ± 12.73 s *vs*. 275.05 ± 14.47 s, paired T-test, p < 0.05), demonstrating that the GRAM model was better than the TREM model for the proposed BBI system.

As shown in Fig. 3(b), the average turning accuracy of all rat cyborgs dropped approximately 15% at the first session of brain control stage 2 compared to that in the manual control stage. The turning accuracy then gradually increased back to 98.08 ± 2.31% at the last session in stage 2, indicating that the rat cyborgs could quickly be accustomed to the transition of different control styles. The drop of the fourth session was most likely due to the poor performance (81.67 ± 5.44%) of one rat cyborg. When the brain control model changed from GRAM at stage 2 to TREM at stage 3, the turning accuracy slightly dropped to 90.35 ± 5.03% in the first session of stage 3 and then generally increased across the remainder of the last stage. The group level of turning accuracy on average for stage 2 and 3 was 91.75 ± 3.85% and 93.32 ± 1.73%, respectively (stage 2 *vs*. stage 3, paired T-test, p > 0.05). Overall, the turning accuracy of stage 2 and stage 3 demonstrated stable behavior results of brain control on rat cyborgs at the group level.

**Figure 4:**
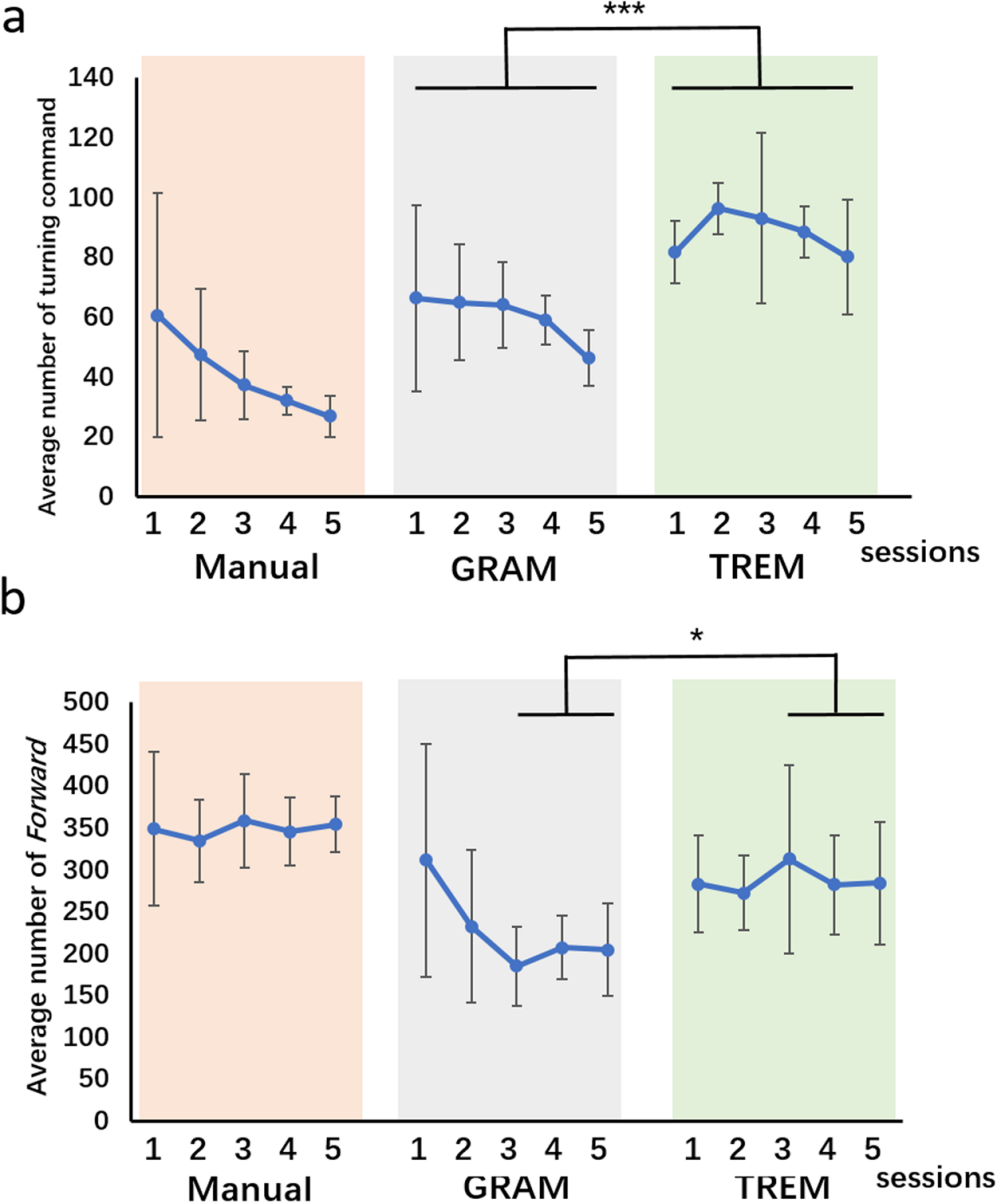
(**a**) Average number of turning instructions for all the rat cyborgs across all the sessions and a comparison of the group-level number of turning instructions between different stages. (**b**) Average number of Forward instructions for all the rat cyborgs across all sessions and a comparison of the group-level number of Forward instruction between different stages. ***indicates p < 0.01, *indicates p < 0.05, paired T-test.

We further analyzed the sending number of different instructions among the three stages. Figure 4(a) shows the average number of *Left* and *Right* turning instructions to complete an experimental run across sessions of all the rat cyborgs tested. Theoretically, the minimum number of turning instructions given in a 100% accuracy run is 16, which can hardly be reached even by experienced manual control. Compared with the GRAM-based and the TREM-based brain control stages, the group-level number of turning instructions were 60.15 ± 7.33 and 87.98 ± 56.30 (stage 2 *vs*. stage 3, paired T-test, p < 0.01), respectively. Thus, more turning instructions were needed to steer the rat cyborg with TREM-based brain control. Since the number of turning instructions was largely affected by the accuracy of the instructions, the extra instructions in TREM were most likely used to compensate the effect of wrong turning behavior. As we mentioned above, instructions given with a proper timing contributed to fewer mistakes; therefore, the lower number of turning instructions in the GRAM-based brain control stage demonstrated that there was less error turning correction in GRAM-based stage 2 than in TREM-based stage 3.

As shown in Fig. 4(b), the group level average of *Forward* instructions across the sessions of GRAM-based and TREM-based brain control was 228.14 ± 44.44 and 286.70 ± 13.57, respectively. The statistical analysis indicated that the sending number of *Forward* instructions had no significant difference (stage 2 *vs*. stage 3, paired T-test, p = 0.09) between the two brain control stages. This may be due to the large fluctuation in the first two sessions of stage 2, which might have been caused by the transition from manual control to brain control. On one hand, the brain-control manipulators needed to gain experiences in controlling rats. On the other hand, the rat cyborgs also needed time to get adapted to new controlling strategy, especially the different stimulation timing and frequency from manual control. When only the later three sessions of stage 2 and stage 3 were compared, the sending *Forward* instruction did show a significant difference (later three sessions, stage 2 *vs*. stage 3, paired T-test, p = 0.03). This result demonstrated that the TREM-based brain control model requires more *Forward* instructions for the rat cyborgs to complete the same turning tasks. The reason for more *Forward* instructions with the TREM-based brain control model was the rat cyborgs had a worse performance with the TREM model and required more turning and forward instructions to correct the wrong behavior.

**Figure 5:**
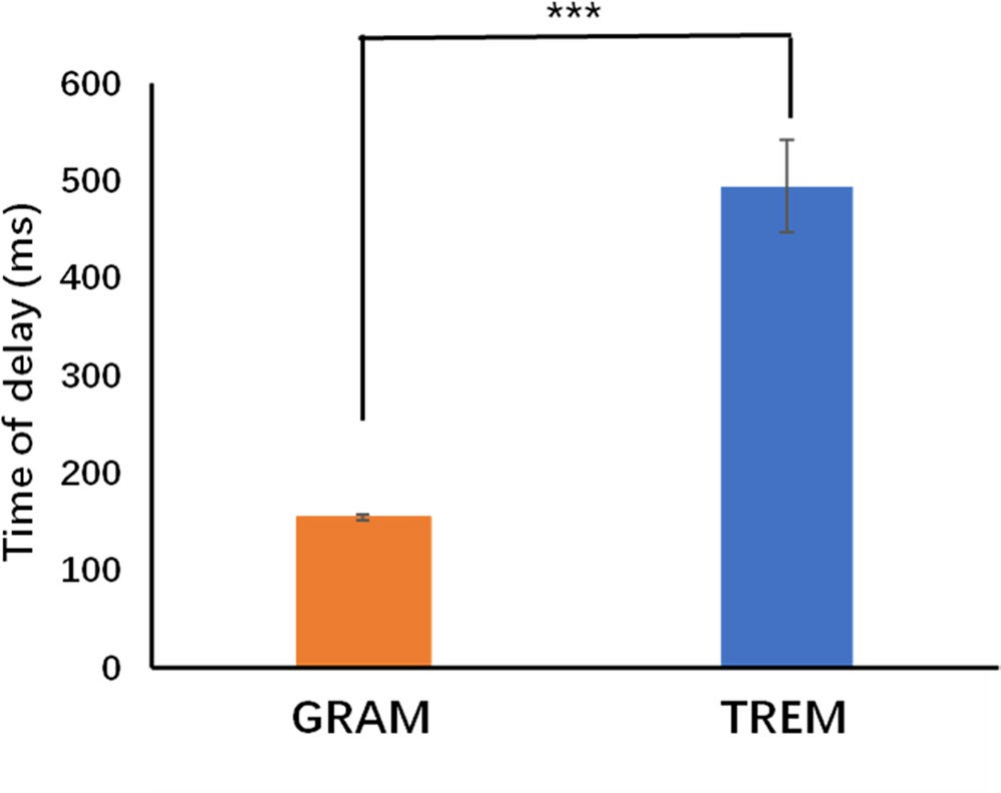
The delay between the start of decoding result output and the instruction generation refers to the thresholds for GRAM and TREM. ***indicates p < 0.01, T-test.

To explain the different performances of GRAM- and TREM- based brain control strategies, we also calculated the short delays occurred between decoding result output from EEG device and instructions generated by two different control models. Our results showed a nearly 70% reduction of instruction generation delay with GRAM (155.01 ± 3.10 ms) compared to TREM (494.70 ± 47.22 ms) (Shown in Fig. 5). Turning instructions were thus generated and sent much quicker after the motor imagery with the GRAM model, which ensured less wrong turning behavior of the rat cyborgs and better turning performance.

**Figure 6:**
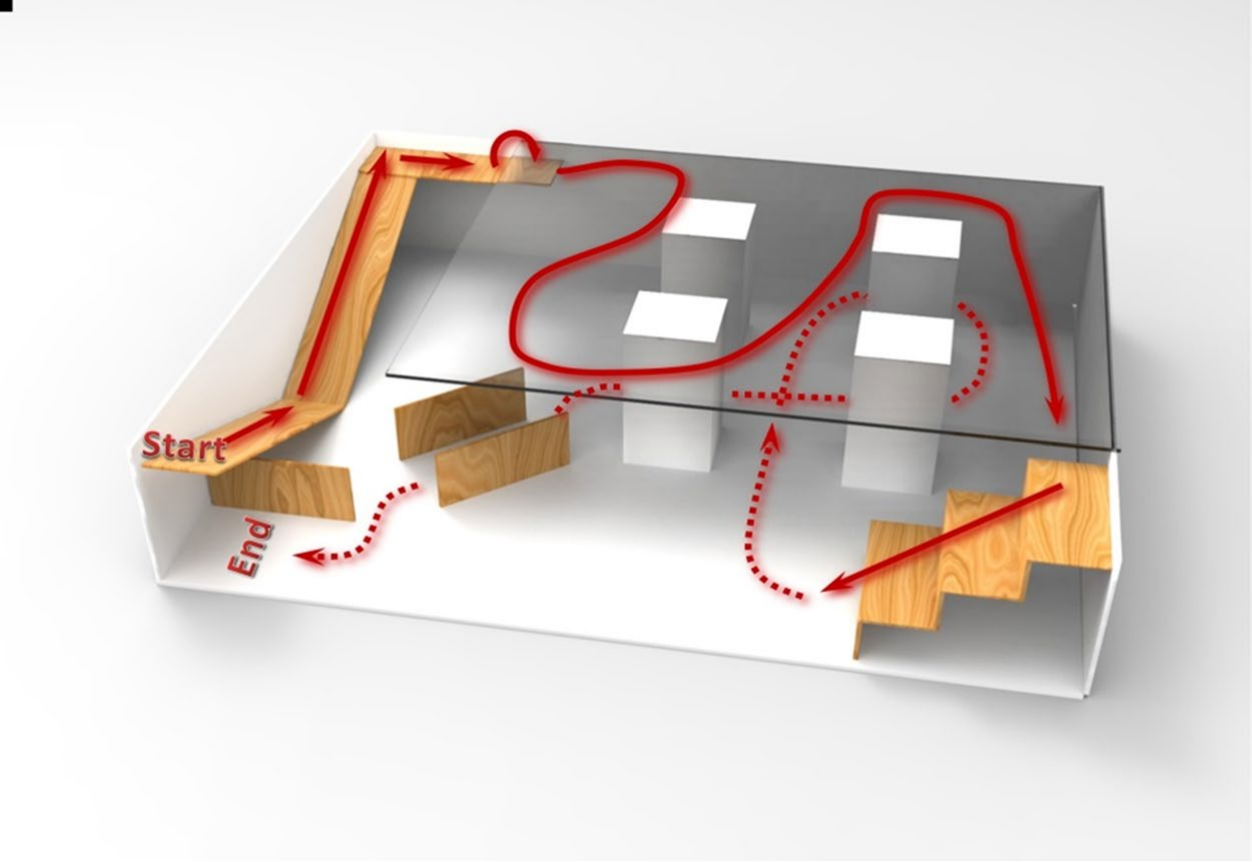
The rat cyborg was navigated by human brain control in a more complex maze (see more details in Supplementary Video 1). The three-dimensional maze was more complicated, consisting of a start point and an end point, slops and stairs for climbing and descending, a raised platform with a height of half a meter, pillars to be avoided and a tunnel to be passed through. The rat cyborgs were asked to complete the navigation task along the preset route (red arrowed) within 5 minutes.

The BBI system was further tested in a maze of higher complexity to test its applicability and stability. The rats were asked to complete a series of preset navigation tasks such as climbing and descending steps, turning left or right, and going through a tunnel in a three-dimensional maze under control of the BBI system. When the rat went into a wrong direction or turned into an unexpected route, the manipulator needed to guide the rat back to the correct route (Fig. 6, see more details in Supplementary Video 1). 5 minutes completion time for each run was limited as the criterion for evaluating success rate. A successful run was defined as the rat cyborgs finish all of preset navigation tasks following the route within the limited time. All rats participated in turning tasks were tested with the optimized GRAM-based brain control model in the maze task. The rats all performed well with high success rate in 10 consecutive tests (Table 1).

**Table 1:** Success rate of brain control in the complex maze.

Rat cyborg	Success	Total	Success rate
A01	8	10	80%
A02	9	10	90%
A03	8	10	80%
A04	9	10	90%
A05	10	10	100%
A06	10	10	100%
**Average**	**9**	**10**	**90%**

## Discussion

Our study demonstrated the feasibility of cultivating an information pathway between a human brain and a rat brain. With our BBI system, a rat cyborg could accurately complete turning and forward behavior under the control of a human mind, and could perform navigation tasks in a complicated maze. Our work extended and explored the further possibility of functional information transmission from brain to brain. Unlike mechanical robots, the rat cyborgs have self-consciousness and flexible motor ability, which means the rat cyborgs will have unexpected movements depending on their own will during the control period. The BBI system should thus be designed with high instantaneity and real-time feedback for a better control effect. Previous brain-to-brain systems have mainly been based on the SSVEP paradigm^15,16^. In the SSVEP paradigm, the manipulators must switch their attention between the feedback screen and the flickers. However, rat cyborgs move quickly and require a minimum frequency of *Forward* instructions above 3 Hz. It is thus difficult for the human manipulator to send a high frequency of *Forward* instructions and simultaneously watch the locomotion of the rat cyborgs in the feedback screen. Compared with previous works^15,16^, we used motor imagery and eye blink as manipulative protocols and provided real-time visual feedback of the rat cyborg, which is comparably more viable and avoids the visual fatigue of the manipulators. In addition, during the rat cyborgs brain control experiments, the overall performance was influenced by several major factors:

1. The accuracy of instructions. The decoding correctness of motor imagery and the appropriate timing of control instructions influence the control performance the most. Furthermore, the instruction should be sent with high instantaneity, especially when an unexpected mistake occurs. In our brain control sessions, the correctness mainly depended on the threshold value and the timeliness of triggering instruction determined by the control models. The better performance (less CPT and number of turning and forward instructions) for GRAM-based BBI is most likely due to less delay between the start of the decoding results and the release of instructions. Comparatively, the longer delay occurred in TREM may probably contribute to a longer CPT, which in turn resulted in greater amount of instructions needed to complete the task. Besides, the longer delay in the TREM model also leads to obstruction of motor imagery. The manipulators reported that the delay of instruction release during TREM brain control could not readily be perceived. Although the manipulators tried to begin imagery in advance, it was difficult to decide the concrete timing and difficult to operate when instructions were needed to be released over a short period. In contrast, with the short response duration in GRAM, the manipulators were able to start motor imagery at the optimal instruction-receiving time, and switching between *Left* and *Right* instructions was much easier.
2. Adaption of the manipulators to brain control task. The mental status of a manipulator can be influenced by disturbance, such as environmental noise, and fatigue caused by long-duration imagery. The ability to overcome these could be improved after several practice sessions. The noninvasive EEG-based BMI used in this study translates the sensorimotor rhythms detected in the bilateral motor areas to the control signal for the rat cyborg. This is not intuitive to the manipulators at the beginning of the experiment, but becomes more intuitive as the experiment goes on. The manipulators gradually learn what instruction should be sent and when their imagery should begin according to the movements and locations of the rat cyborg, thereby cultivating a tacit understanding between the human and the rat cyborg. The stable level of performance seen in the latter stage 2 and stage 3 indicates this mutual adaption.
3. The inherent adaptive ability of rat cyborgs. Rat cyborgs possess an inherent adaptive ability to their environment and the control method. The overall decrease of average CPT in the manual control stage indicates the adaption of rat cyborgs to the control instructions. The variation trend of each line indicates the various adaption abilities among rat cyborgs. Intriguingly, the final CPT of each rat cyborg reached a similar level. It is likely that all of the rat cyborgs adapted to the same control pattern of the operator. In addition, the rat cyborgs can also adapt to the changes of instruction release due to their excellent learning ability. The results showed that the performance was adversely affected by changes in the control mode (stage 1, session 5 *vs*. stage 2, session 1 and stage 2, session 5 *vs*. stage 3, session 1 in Fig. 3(a,b)) but subsequently stabilized. The decrease in the turning accuracy from stage 1 to stage 2 was much sharper than the change from stage 2 to stage 3. This may be because the control pattern is more distinct between manual control and brain control. While between different brain control stages, the manipulator’s control pattern was not likely to dramatically alter.
4. In conclusion, our findings suggest that computer-assisted BBI that transmits information between two entities is intriguingly possible. The control model proposed here could transfer the decoding results of motor imagery-based EEG-BMI to other external devices with remarkable instantaneity. In the future, error-related potentials (ErrPs)^21^ could be used to detect false generated instructions, thereby eliminating the wrong instructions before sending them to the rat cyborgs. Furthermore, information flow will be made bidirectional and communicative between two human individuals.

## Methods

### Participants and ethics statement

Six rats were engaged in this study. All methods were carried out in accordance with the National Research Council’s Guide for the Care and Use of Laboratory Animals. All experimental protocols were approved by the Ethics Committee of Zhejiang University, China. Informed consent was obtained from all manipulators.

### Rat cyborg preparation

The rat cyborg system had long been developed in our previous research work. Briefly, bipolar stimulating electrodes were made from pairs of insulated nichrome wires (65 μm in diameter), with a 0.5 mm vertical tip separation. Microelectrodes were implanted into the rat’s brain for the control of their locomotion. Two pairs of electrodes were implanted in the bilateral medial forebrain bundle (MFB)^22^ for virtual reward stimulation and instruction of forward moving. The other two pairs of electrodes were implanted symmetrically in both sides of the whisker barrel fields of somatosensory cortices (SIBF)^23^ for turning cue stimulation. The rats were allowed to recover from the surgery for one week before the experiments. Once recovered, the rat cyborgs were first trained to correlate the stimulations with the corresponding locomotion behaviors^17^. The parameters of the electrical stimulation that were sent into the rat’s brain were based on our previous works^24^, which can activate appropriate behavior but avoid seizures even after a long duration of stimulation. During the training and control sessions, electrical stimulations were delivered through a wireless microstimulator mounted on the rat’s back. Control instructions were given by operators with a computer program wirelessly connected to the microstimulator through Bluetooth.

### Decoding in the BBI

A commercial EEG device, Emotiv EPOC (Emotiv Inc., USA)^25^ was used in this study for EEG data recording. EEG data were acquired with a 14-channel neuroheadset, with all electrode impedances kept below 10 kΩ. During the brain control experiments, the EEG signals were sampled at the rate of 256 Hz. The recorded data were then wirelessly transmitted to a host computer through Bluetooth and further processed with the help of Emotiv SDK. Through trained imagination, the manipulators learned to modulate their sensorimotor rhythm amplitude in the upper mu (10–14 Hz) frequency band^26,27^. The power spectrum of left and right composition was then obtained as the intensity of motor imagery by common spatial pattern (CSP)^28^, i.e., *x*_*L*_(*t*) and *x*_*R*_(*t*), respectively. Details of the common spatial pattern filter are described as follows:

Let X_*R*_ and X_*L*_ denote the preprocessed EEG during right- or left-hand movements with dimensions *N* × *T*, where N is the number of channels and T is the number of samples per channel. The common spatial pattern filter is acquired as follows:

1. Calculate the normalized channel covariance of X_*R*_ and X_*L*_ as:
  1 $${C}_{L}=\frac{{\rm{cov}}({{\rm{X}}}_{L})}{{\rm{trace}}({{\rm{X}}}_{{L}}{{\rm{X}}}_{L}^{T})}$$
  2 $${C}_{R}=\frac{{\rm{cov}}({{\rm{X}}}_{R})}{{\rm{trace}}({{\rm{X}}}_{R}{{\rm{X}}}_{R}^{T})}$$
2. Average the *C*_*L*_ and *C*_*R*_ on all of the left- and right-hand movement EEG trials; the composite spatial covariance is:
  3 $$C=\overline{{C}_{L}}+\overline{{C}_{R}}$$
3. Perform eigenvalue decomposition on the composite spatial covariance, where:
  4 $$C={U}_{0}{\rm{\Sigma }}{U}_{0}^{T}$$
4. Perform whitening transform on $\overline{{C}_{L}}$ and $\overline{{C}_{R}}$, and the transformed spatial covariance matrixes are:
  5 $${S}_{L}=P\overline{{C}_{L}}{P}^{T}$$
  6 $${S}_{R}=P\overline{{C}_{R}}{P}^{T}$$
  where,
  7 $$P={{\rm{\Sigma }}}^{-1/2}{U}_{0}^{T}$$
5. Perform eigenvalue decomposition on the transformed spatial covariance matrix, where:
  8 $${S}_{L}={U}_{L}{{\rm{\Sigma }}}_{L}{U}_{L}^{T}$$
  9 $${S}_{R}={U}_{R}{{\rm{\Sigma }}}_{R}{U}_{R}^{T}$$
  (Note that Σ_*L*_ + Σ_*R*_ must be an identity matrix);
6. The eigenvectors corresponding to the largest eigenvalue in Σ_*L*_ and Σ_*R*_ are chosen to calculate the common spatial pattern filters for right- and left-hand movements, which can be written as:
  10 $$S{F}_{L}={U}_{L}(i|{{\rm{argmax}}}_{i}\,{{\rm{\Sigma }}}_{L}(i))P$$
  11 $$S{F}_{R}={U}_{R}(j|{{\rm{argmax}}}_{j}\,{{\rm{\Sigma }}}_{R}(j))P$$
7. Let *x*(*t*) be the preprocessed EEG signal recorded in movement imaginary application, the intensity of left- and right-hand movement imagery can be given as:
  12 $${x}_{L}(t)=S{F}_{L}x(t)$$
  13 $${x}_{R}(t)=S{F}_{R}x(t)$$
8. Finally, calculate the power spectral density of *x*_*L*_(*t*) and *x*_*R*_(*t*), and aggregate the band power within the overlapping window length of k.

14 $${{\rm{B}}}_{L}(t)=\sum _{t-k}^{t}P({x}_{L}(t))$$

15 $${{\rm{B}}}_{R}(t)=\sum _{t-k}^{t}P({x}_{R}(t))$$

where $P(x(t))$ indicates the power spectral density of *x*(*t*). The intensity of motor intent was then mapped to a value ranged from 0 to 1, and the normalized *B*(*t*) was used as the input of the control model.

### Set up of BBI system

As the BBI system consisted of a noninvasive EEG-based BMI and a rat cyborg system, a controlling program written in Visual C++ was applied to acquire EEG raw data from Emotiv SDK, generate instructions with the control models and trigger the release of instructions to the rat cyborg. The locomotion and location of the rat cyborg in the entire experimental scene was video captured by a top-viewed camera and visually delivered back to the manipulators on an LCD screen in real time. The decoding results of motor imaginary were relayed using a flashing instruction feedback panel that was integrated in the bottom of the LCD by an OpenCV (Open Source Computer Vision Library, http://opencv.org)-based self-written program. The EEG decoding results and motor control instructions were recorded with a J2EE (Java 2 Platform Enterprise Edition)-based program for further analysis.

### Control models for BBI

The inputs of the control model included the decoding results of *Left* or *Right* motor imagery and eye blink detection. The collected EEG signals were projected by a common spatial pattern (CSP) spatial filter. Next, the power spectrum of left and right composition was obtained as the intensity of motor imagery, i.e., *x*_*L*_(*t*) and *x*_*R*_(*t*), respectively. Eye blink, *x*_*F*_(*t*), was detected when the EEG signal $\overrightarrow{(E(t))}$ amplitude of channels near the eyes exceeds a threshold $\overrightarrow{{\theta }_{EOG}}$.

16 $${x}_{F}(t)=\{\begin{array}{ll}1, & \overrightarrow{E(t)}\ge \overrightarrow{{\theta }_{EOG}}\\ 0, & Otherwise\end{array}$$

The output of the control model was a control signal for the microelectrical stimulations. *x*_*L*_(*t*), *Y*_*R*_(*t*) and *Y*_*F*_(*t*) represent the *Left*, *Right* and *Forward* instructions, respectively.

For the safety of the rat cyborgs, instructions should be sent under the following rule: If two instructions were presented continuously, the latter instruction would only be sent when the time interval was larger than a predefined threshold Δ*T*. Adjacent instructions were defined as tuples <C1, C2>, C1, C2 ∈{*Left*, *Right*, *Forward*}. Five out of nine types of tuples were restricted, namely, Δ*T*_<*F*,*F*>_, Δ*T*_<*L*,*L*>_, Δ*T*_<*R*,*R*>_, Δ*T*_<*L*,*F*>_ and Δ*T*_<*R*,*F*>_. These five were determined by the number distribution of the interval for each tuple based on the manual control sequence record. To guarantee the proper reaction, the level of excitement and the safety of the rat cyborgs, the intervals of Δ*T*_<*F*,*F*>_, Δ*T*_<*L*,*L*>_, Δ*T*_<*R*,*R*>_, Δ*T*_<*L*,*F*>_ and Δ*T*_<*R*,*F*>_ for brain control were set to be 200 ms, 500 ms, 500 ms, 350 ms and 350 ms, respectively, according to our previous work^17^. The minimum time interval was not restricted for F-L, F-R, R-L and L-R because the manipulator needed to send the first turning command as quickly as possible.

We defined *n* = 0, 1, … as the n-th generation of instruction, and *t*_*L*_(*n*), *t*_*R*_(*n*) and *t*_*F*_(*n*) were the times that an instruction occurred. Initially, *t*_*L*_(*n*), *t*_*R*_(*n*) and *t*_*F*_(*n*) were equal to 0 (n = 0). The generation of *Forward* was the same for the two models, as described below:

17 $${Y}_{F}(t)=\{\begin{array}{ll}1, & t\in \{{{\rm{t}}}_{F}|\begin{array}{l}{t}_{F}(n)-{t}_{F}(n-1)\ge {\rm{\Delta }}{T}_{ < F,F > },\\ {t}_{F}(n)-{t}_{L}(n-1)\ge {\rm{\Delta }}{T}_{ < L,F > },\\ \begin{array}{c}{t}_{F}(n)-{t}_{R}(n-1)\ge {\rm{\Delta }}{T}_{ < R,F > }\\ and\,{x}_{F}({{\rm{t}}}_{F})=1\end{array}\end{array}\}\\ 0, & otherwise\end{array}$$

**Figure 7:**
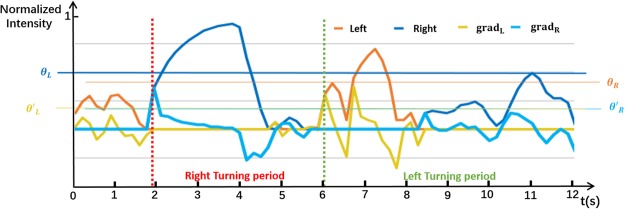
Samples of decoding results and their corresponding gradients of motor imagery in a preliminary experiment. The blue curve is the result of right imagery (Right) and the orange curve is the result of left imagery (Left). During the right turning period shown in the figure, only right imagery occurred, while in the left turning period, both left and right results appeared. The right decoding results were deemed to be caused by noise. In addition, the left decoding results appearing in the blank period (no imagination) are regarded as noise as well. The grad_L_ (yellow) and grad_*R*_ (light blue) curves represent the left and right gradient of corresponding decoding results, respectively. *θ*_*L*_ and *θ*_*R*_ are the optimal thresholds for left and right motor imagery in TREM. For GRAM, the optimal thresholds are *θ*_*L*_ and *θ*_*R*_.

Two models (Fig. 1(b)) for generating *Left* and *Right* instructions were proposed. One was called the thresholding model (TREM), in which the instructions were generated when the decoding results exceeded a threshold (*θ*). The other model was the gradient model (GRAM), in which the instructions were generated when the gradient value between two decoding results transcended a threshold (*θ*′). The thresholds were used to differentiate the decoding results attributed to real intention or noise. Figure 7 demonstrates typical decoding results of a left and right imagery and their corresponding gradients.

#### Thresholding Control Model

For TREM, controlling impulses were generated when the intensity of left or right exceeded a threshold *θ*. A turning instruction was generated if *x*_*L*_(t) > *θ*_*L*_ or *x*_*R*_(t) > *θ*_*R*_. Therefore, the function of TREM is described as follows:

18 $${Y}_{L}(t)=\{\begin{array}{ll}1, & t\in \{{t}_{L}|{t}_{L}(n)-{t}_{L}(n-1)\ge {\rm{\Delta }}{T}_{ < L,L > }\,and\,\begin{array}{c}{x}_{L}({t}_{L})\end{array}\ge {\theta }_{L}\}\\ 0, & otherwise\end{array}$$

19 $${Y}_{R}(t)=\{\begin{array}{ll}1, & t\in \{{t}_{R}|{t}_{R}(n)-{t}_{R}(n-1)\ge {\rm{\Delta }}{T}_{ < R,R > }\,and\,\begin{array}{c}{x}_{R}({t}_{R})\end{array}\ge {\theta }_{R}\}\\ 0, & otherwise\end{array}$$

#### Gradient Control Model

Although the threshold in TREM could differentiate the decoding results attributed to real intention or floating background noise, the delay between the start of the decoding results and the generation of instruction was too long. We proposed an improved model, GRAM, that outperformed in both differentiation and instantaneity. For GRAM, instructions were generated when the gradient value between two decoding windows transcended a threshold *θ*′. The gradient value was calculated as follows:

20 $$Grad\begin{array}{c}x(t)\end{array}=x(t)-x(t-1)$$

A turning instruction was generated if Grad *x*(*t*) > *θ*′. Accordingly, the function of GRAM is described as follows:

21 $${Y}_{L}(t)=\{\begin{array}{ll}1, & t\in \{{t}_{L}|{t}_{L}(n)-{t}_{L}(n-1)\ge {\rm{\Delta }}{T}_{ < L,L > }\,and\,Grad\begin{array}{c}{x}_{L}({t}_{L})\end{array}\ge {\theta ^{\prime} }_{L}\}\\ 0, & otherwise\end{array}$$

22 $${Y}_{R}(t)=\{\begin{array}{ll}1, & t\in \{{t}_{R}|{t}_{R}(n)-{t}_{R}(n-1)\ge {\rm{\Delta }}{T}_{ < R,R > }\,and\,Grad\begin{array}{c}{x}_{R}({t}_{R})\end{array}\ge {\theta ^{\prime} }_{R}\}\\ 0, & otherwise\end{array}$$

The thresholds *θ* and *θ*′ were decided prior to the implementation of brain control. To ascertain the optimal threshold, a preliminary experiment was conducted. The manipulators were asked to complete three rounds of eight motor imagery tasks. Intents were decoded in real time, and the decoding results were recorded. The best threshold was determined with a receiver operating characteristic (ROC) curve.

## Supplementary information

Supplementary video 1

Supplementary video 2-Rat cyborg with Forward instructions only

Supplementary video 3-Rat cyborg without instructions

## Data Availability

The datasets generated during and/or analyzed during the current study are available from the corresponding author on reasonable request.
